# Single-plane versus real-time biplane approaches for ultrasound-guided central venous catheterization in critical care patients: a randomized controlled trial

**DOI:** 10.1186/s13054-023-04635-y

**Published:** 2023-09-23

**Authors:** Ying-Ying Li, Yi-Hao Liu, Lin Yan, Jing Xiao, Xin-Yang Li, Jun Ma, Li-Gang Jia, Rui Chen, Chao Zhang, Zhen Yang, Ming-Bo Zhang, Yu-Kun Luo

**Affiliations:** 1 From the Medical School of Chinese PLA, No. 28 Fuxing Rd, Haidian District, Beijing, 100853 China; 2 Department of Ultrasound, First Medical Center, Chinese PLA General Hospital, No. 28 Fuxing Rd, Haidian District, Beijing, 100853 China; 3 Department of Ultrasound, People’s Hospital of Torch Development Zone, Zhongshan, China

**Keywords:** Ultrasound, Central venous catheterization, Real-time biplane imaging, Internal jugular vein, Femoral vein

## Abstract

**Background:**

Critical care patients often require central venous cannulation (CVC). We hypothesized that real-time biplane ultrasound-guided CVC would improve first-puncture success rate and reduce mechanical complications. The purpose of this study was to compare the success rate and safety of single-plane and real-time biplane approaches for ultrasound-guided CVC.

**Methods:**

From October 2022 to March 2023, 256 participants with critical illness requiring CVC were randomized to either the single-plane (*n* = 128) or biplane (*n* = 128) ultrasound-guided cannulation groups. The success rate, number of punctures, procedure duration, incidence of catheterization-related complications, and confidence score of operators were documented.

**Results:**

The central vein was successfully cannulated in all 256 participants (163 [64%] man and 93 [36%] women; mean age 69 ± 19 [range 13–104 years]), including 182 and 74 who underwent internal jugular vein cannulation (IJVC) and femoral vein cannulation (FVC), respectively. The incidence of successful puncture on the first attempt was higher in the biplane group than that in the single-plane group (91.6% vs. 74.7%; relative risk (RR), 1.226; 95% confidence interval (CI), 1.069–1.405; *P* = 0.002 for the IJVC and 90.9% vs. 68.3%; RR, 1.331; 95% CI, 1.053–1.684; *P* = 0.019 for the FVC). The biplane group was also associated with a higher first-puncture single-pass catheterization success rate (87.4% vs. 69.0% and 90.9% vs. 68.3%), fewer undesired punctures (1[1–1(1–2)] vs. 1[1–2(1–4)] and 1[1–1(1–3)] vs. 1[1–2(1–4)]), shorter cannulation time (205 s [162–283 (66–1,526)] vs. 311 s [243–401 (136–1,223)] and 228 s [193–306 (66–1,669)] vs. 340 s [246–499 (130–944)]), and fewer immediate complications (10.5% vs. 28.7% and 9.1% vs. 34.1%) for both IJVC and FVC (all *P* < 0.05).

**Conclusion:**

Real-time biplane imaging of ultrasound-guided CVCs offers advantages over the single-plane approach for critically ill patients.

*Trial registration*: This prospective RCT was registered at Chinese Clinical Trial Registry (ChiCTR2200064843). Registered 19 October 2022.

**Supplementary Information:**

The online version contains supplementary material available at 10.1186/s13054-023-04635-y.

## Background

Central venous catheterization (CVC), which can be lifesaving for critically ill patients, is frequently performed to aid the intravenous administration of fluid resuscitation, allow safe intravenous administration of medication, facilitate hemodialysis, and help in the monitoring of hemodynamic variables in the intensive care unit (ICU) [1, 2]. More than 15 million CVCs are performed each year in the United States [3], and 70 CVCs per 100 patient-days are performed in the European ICUs [4]. Central venous access is commonly attempted through the internal jugular vein (IJV), subclavian vein, and femoral vein (FV). IJV and FV are the primary choices, as recommended by the guidelines, both in elective and emergency settings [5–10] because of their advantage of being less frequently associated with mechanical complications [8, 11–15]. Ultrasound (US) guidance has been extensively studied to reduce the number of complications and improve the safety and quality of CVCs [16–19]; therefore, US-guided techniques have become widely accepted and are recommended by the guidelines for CVCs [7–10, 20–24].

Two different two-dimensional (2D) US techniques employed for CVCs, including out-of-plane and in-plane approaches, are both single-plane display techniques [9, 24, 25]. The out-of-plane method allows for the simultaneous visualization of the vein in relation to the surrounding critical structures but can render the needle tip control difficult [26]. The in-plane method is currently recommended for an in-plane needle insertion with better visualization of the needle throughout its course and depth, which can lead to more precise needle tip control [9, 20]; however, it can be challenging to perform owing to certain anatomical limitations, such as short neck, and arterial puncture can occur without visualization. No rigorous conclusions regarding the clinical value of the different approaches can be drawn because each approach has its own set of advantages and disadvantages.

The x-plane technique, a real-time three-dimensional (3D) imaging technique, uses a matrix array probe (3–14 MHz) that takes advantage of the strengths and overcomes the limitations of previous approaches, allowing for simultaneous imaging of both transverse and longitudinal views without rotating the probe. Thus, the x-plane approach creates a comprehensive picture of both the needle trajectory and vein with its surrounding structures. To our knowledge, only one study has compared US-guided out-of-plane and biplane imaging for internal jugular catheterization (IJVC) with a low-frequency transducer [28]; however, no study has compared the in-plane and the x-plane techniques. Therefore, this present study aimed to compare the success rates and safety of single- and x-plane US-guided CVCs (IJVC and femoral vein catheterization (FVC)) in critically ill patients.

## Materials and methods

### Study population

**Fig. 1:**
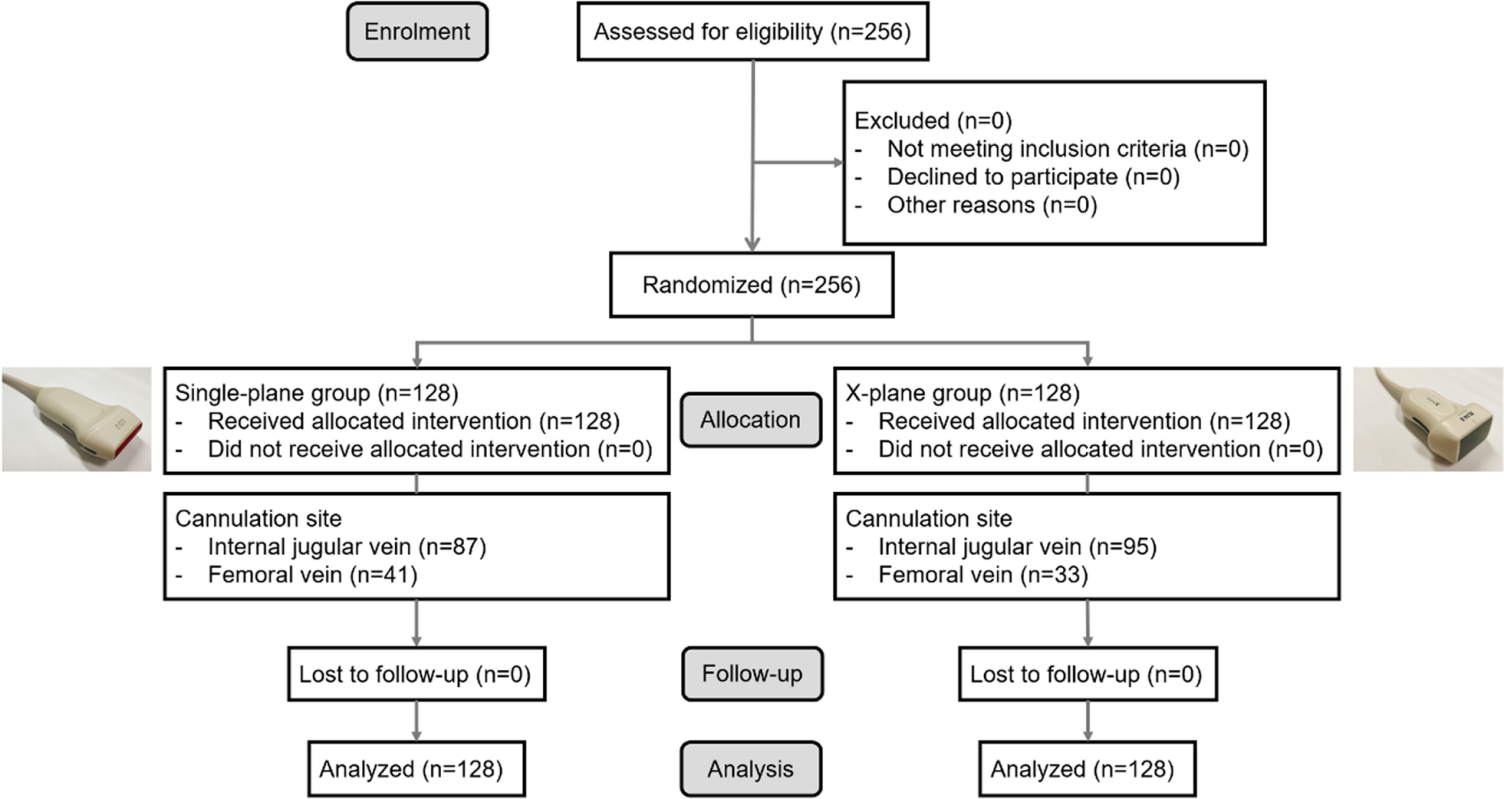
CONSORT flow diagram of the study

This prospective, single-center, randomized clinical trial was conducted at The First Medical Center of Chinese PLA General Hospital. The study protocol was approved by the local Ethics Committee and was registered at Chinese Clinical Trial Registry. Written informed consent was obtained from each patient diagnosed with a critical illness requiring CVC between October 2022 and March 2023. A flow diagram of the study is shown in Fig. 1.

### Methods

All CVC procedures were performed for clinical reasons. The decision to perform CVC, either IJVC or FVC, was made by the physician on duty and was not involved in the study. Patients were randomized to the x-plane or single-plane approaches using means of a computer-generated random number table. Vital signs and coagulation indices were recorded.

Each cannulation was performed at the patient’s bedside by one of five sonographers with three months of experience in US-guided CVC. They had similar experience with x-plane and single-plane cannulations (> 10 procedures/month; 50% in the x-plane and 50% in the single-plane). Two-lumen 7F, 20 cm-long CVCs (CS-27702, Arrow Gard Blue®; Teleflex Medical IDA, Ireland) were used for infusion and dual-lumen 13.5F, dialysis catheters (5593240/5594150, Niagara ™; Bard Inc., USA) were used for dialysis.

#### US technique

A Philips (Philips Healthcare, Netherlands) CX50 system equipped with a L12-3 probe and an EPIQ system equipped with a XL14-3 probe were used for single-plane and x-plane CVCs, respectively. The CVCs were performed following previously recommended steps [24]. The primary catheterization site was the right IJV or right FV. The left side was selected in the patients with a thrombus on the right side. The patient was placed in the supine position with a head roll to extend the neck or the leg abducted to expose the puncture site. Conventional 2D-US was used to measure the depth and caliber of the vein, evaluate its patency and compressibility, and identify thrombi in the vein.

The puncture site was prepared using antiseptic solution and protected with sterile drapes, whereas the probe was covered with a sterile sheath using sterile gel outside and inside the sheath. A real-time, single-operator, freehand technique was used. While introducing the needle, the operator held the probe with one hand, and the needle with the other hand, lidocaine 2% was used to anesthetize the puncture site, followed by needle puncture into the vein with the modified Seldinger technique.

The single-plane approach was performed under the guidance of the long-axis view of the vein using in-plane method. The needle was held at 30°–45° angle, oriented in-plane with transducer. Vessel alignment was maintained during the procedure, and the entire length of the needle was visible during progression through the tissues.

Using the x-plane approach, the vein was visualized simultaneously in transverse and longitudinal images. The probe was positioned as the single-plane approach, maintaining the vein in the middle of both the long- and short-axis views. During the procedure, the entire length of the needle was visible in-plane in the long-axis view, and the transient vessel deformation produced by the additional pressure when the tip abutted the venous wall was observed in the short-axis view. Once the vessel wall was penetrated, the deformation disappeared and a hyperechoic dot was displayed within the vein in the short-axis view.

**Fig. 2:**
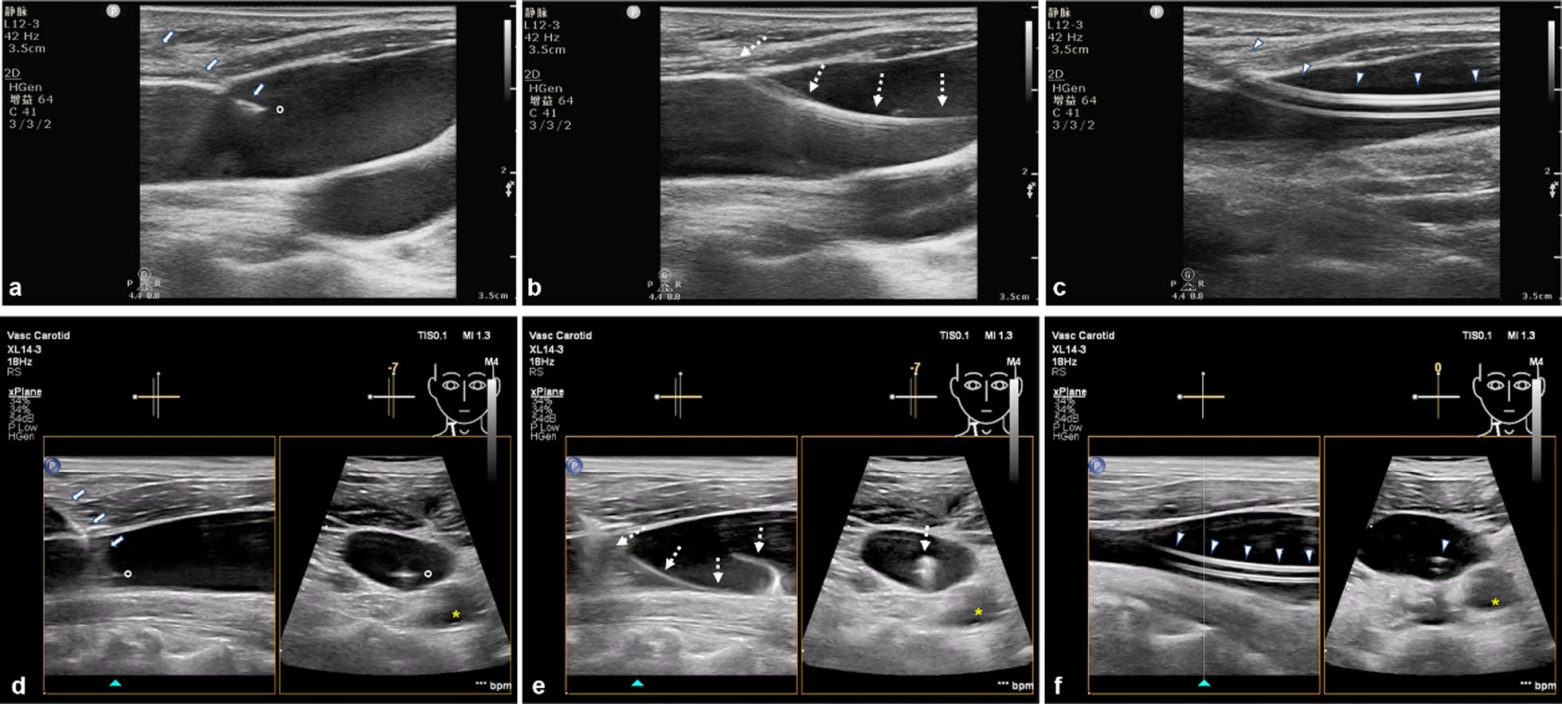
Upper panels show **a** 91-year male underwent the internal jugular vein catheterization using the single-plane approach, with two puncture attempts, with cannulation time of 335 s. Lower panels show a 69-year female underwent the internal jugular vein catheterization using the x-plane approach (simultaneous imaging both longitudinal and transverse views) with cannulation time of 179.6 s with the first-puncture single-pass catheterization success. **a**, **d** represents the needle was punctured into the target vein; **b**, **e** represents the guidewire was inserted into the target vein; **c**, **f** shows that the catheter was located within the vein after catheterization. *White arrows* show the needle track, *white circle* point of the needle, *asterisk* the common carotid artery, *dashed arrows* the guidewire, *white triangle* the catheter

In both single- and x-plane approaches, the presence of the needle in the vein was confirmed by aspiration of blood into the syringe. When the needle tip was inserted into the vessel, the assistant advanced the guidewire. The needle was withdrawn, a dilator was used, and a catheter inserted as usual (Additional file 1: Vedio S1, Fig. 2). An external observer recorded the length of the procedure from the time the probe first touched the sterile field of the patient’s skin to the time the catheter was positioned into the vein. After two failed attempts, the procedure was performed by a superior sonographer. At the end of the procedure, the position of the catheter and presence of complications were assessed using US, while the accurate positioning of the IJVC tip in the superior vena cava was ascertained within 24 h of the procedure using X-ray.

#### Outcome variables

The primary outcome was the first-puncture success rate, defined as the incidence of successful puncture in the target vein at first needle insertion attempt. In addition, we assessed other incidences including first-puncture single-pass catheterization success rate, defined as the incidence of successful catheterization whereby the guidewire, dilator, and catheter were all successfully inserted without any withdrawal for redirection or reinsertion at the first needle puncture attempt and successful final catheterization; number of puncture attempts; puncture time; total catheterization time; complications including immediate mechanical complications such as undesired puncture (multiple skin breach with redirection of the needle), hematoma, posterior venous wall puncture, arterial puncture, pneumothorax, and hemothorax and late complications such as venous thrombosis and central line-associated blood stream infection (CLA-BSI) within 30 days after cannulation; and operator confidence score.

Puncture time was defined as the period between skin penetration by the needle and the first flashback of blood. The total catheterization time was defined as the period between the probe positioning on the patient’s skin and insertion of the catheter. The operator confidence score was graded according to their degree of confidence during the CVC procedure: 1-point, lack of confidence in the first puncture success; 2-point, intermediate grade; 3-point, very confident in the first puncture success.

#### Statistical analysis

The sample size was calculated assuming that the first-needle-puncture success rate was 0.75 in the single-plane group based on previous studies, which reported success rates of 52–85.5% when using single-plane US-guided technique in IJVC and FVC [25, 29–31]. Almost 122 patients per group were required to detect a difference of 0.15 in the first-needle-puncture success rate between the groups, with a dropout rate of 20% (alpha = 0.05; power = 0.80; two-sided test).

Data are expressed as median, interquartile range (IQR), range, count number, or percentage, as indicated. The Kolmogorov–Smirnov test was used to evaluate the normal distribution of continuous variables. Unpaired Student’s *t*-test, Mann–Whitney *U*-test, Chi-square test, or Fisher’s exact test were used where appropriate to identify differences between the two groups for continuous or categorical variables. The probability of lack of complications after CVC positioning was calculated using the Kaplan–Meier product-limit estimator. The log-rank (Mantel-Cox) test was used to evaluate the difference in the probability of lack of complications after grouping for the single-plane versus x-plane approach. Statistical significance was set at a *P* value < 0.05. Statistical analyses were performed using PASS (version 15; NCSS, LLC. Kaysville, Utah, USA), SPSS (version 26.0; Chicago, IL, USA), and GraphPad Prism (version 9.0.0; USA).

## Results

Catheter positioning was possible in all 256 patients using either the single- or x-plane approach. The baseline characteristics and the ultrasonographic characteristics of the study population are summarized in Table 1 and Additional file 2: Table S1, respectively. No significant differences were observed in patient characteristics between the two groups.

**Table 1:** Baseline characteristics of the study population

Characteristics	Single-plane (*n* = 128)	x-Plane (*n* = 128)	*P*
Age (years)	69 (52–84 [13–104])	74 (58–86 [18–99])	0.128
*Sex*			
Male	79 (61.7%)	84 (65.6%)	0.516
Female	49 (38.3%)	44 (34.4%)	
Height (cm)	167 (161–171 [130–183])	168 (160–173 [140–186])	0.999
Body weight (kg)	64 (55–78 [34–120])	63 (55–75 [40–120])	0.625
BMI (kg/m^2^)	23.4 (20.6–27.0 [14.8–38.3])	22.7 (20.4–25.8 [16.0–36.5])	0.420
Temperature (°C)	36.5 (36.3–36.7 [35.8–38.8])	36.5 (36.4–36.7 [35.9–38.9])	0.113
Pulse (bpm)	83 (76–98 [58–133])	86 (75–104 [55–162])	0.388
Respiration (bpm)	18 (18–19 [13–27])	18 (18–19 [12–30])	0.957
Systolic pressure (mmHg)	124 (106–138 [63–187])	124 (114–142 [75–191])	0.217
Diastolic pressure (mmHg)	72 (63–81 [31–110])	74 (66–80 [45–140])	0.455
TT (s)	16.5 (15.8–18.3 [13.6–96.8])	17.0 (16.0–19.0 [13.4–203.0])	0.184
APTT (s)	37.5 (33.1–43.3 [22.0–160.1])	38.6 (34.6–44.0 [24.9–163.3])	0.429
PT (s)	14.6 (13.7–16.2 [11.5–35.4])	15.1 (13.7–16.4 [10.8–116.8])	0.561
PTA (%)	78.0 (63.6–89.0 [20.0–121.0])	76.0 (65.0–86.0 [5.0–127.0])	0.584
INR	1.14 (1.05–1.30 [0.91–3.50])	1.17 (1.07–1.30 [0.89–16.09])	0.583
FIB (g/L)	3.83 (2.87–4.91 [1.26–8.03])	4.02 (2.88–5.77 [0.60–10.67])	0.116
D–Dimer (μg/ml)	2.58 (1.31–5.09 [0.14–20.00])	2.57 (1.18–4.77 [0.26–20.00])	0.889
Platelets (× 10^3^/μL)	186 (130–244 [9–402])	181 (111–245 [9–500])	0.970
*Reasons for catheterization (n)*			
Infusion	102 (79.7%)	106 (82.8%)	0.522
Dialysis	26 (20.3%)	22 (17.2%)	
*Cannulation site*			
IJV	87 (68.0%)	95 (74.2%)	0.270
FV	41 (32.0%)	33 (25.8%)	
Side of catheterization (n)			
Left	15 (11.7%)	23 (18.0%)	0.160
Right	113 (88.3%)	105 (82.0%)	

*BMI* Body mass index; *TT* Thrombin time; *APTT* Activated partial thromboplastin time; *PT* Prothrombin time; *PTA* Prothrombin activity; *INR* International normalized ratio; *FIB* Fibrinogen; *IJV* Internal jugular vein; *FV* Femoral vein

The procedural data are summarized in Table 2. The primary outcome, the incidence of first-puncture success was higher in the x-plane group than that in the single-plane group [91.6% vs. 74.7%; relative risk (RR), 1.226; 95% confidence interval (CI), 1.069–1.405; *P* = 0.002; and 90.9% vs. 68.3%; RR, 1.331; 95% CI, 1.053–1.684; *P* = 0.019 for IJVC and FVC, respectively]. In addition, the number (median [IQR (range)]) of puncture attempts was lower in the x-plane group than that in the single-plane group (1[1–1(1–2)] vs. 1[1–2(1–4)]; *P* < 0.001 and 1[1–1(1–3)] vs. 1[1–2(1–4)]; *P* = 0.029 for IJVC and FVC, respectively). The total CVC time in seconds was shorter in the x-plane group than in the single-plane group (205 [162–283 (66–1,526)] vs. 311 [243–401 (136–1,223)]; *P* < 0.001 and 228 [193–306 (66–1,669)] vs. 340 [246–499 (130–944)]; *P* < 0.001 for IJVC and FVC, respectively), as well as the puncture time. A higher confidence score of the operator during the CVC procedure after the x-plane guidance (2 [2–3 (1–3)] vs. 2[1–2(1–3)]; *P* = 0.008 and 3 [2–3 (1–3)] vs. 2[2–2(1–3)]; *P* < 0.001 for IJVC and FVC, respectively) was statistically significant.

**Table 2:** Comparisons between single-plane and x-plane group for outcomes of central venous catheterization

	Single-plane	x-Plane	*P*	RR	95% CI
IJVC	*n* = 87	*n* = 95			
First-puncture success	65 (74.7%)	87 (91.6%)	0.002	1.226	1.069–1.405
First-puncture single-pass catheterization success	60 (69.0%)	83 (87.4%)	0.003	1.267	1.079–1.487
Successful final catheterization	87/87 (100%)	95/95 (100%)	-		
Puncture attempts (n)	1 [1–2 (1–4)]	1 [1–1 (1–2)]	< 0.001		
Puncture time (s)	70 [45–143 (18–1,079)]	43 [23–100 (9–802)]	< 0.001		
Total catheterization time	311 [243–401 (136–1,223)]	205 [162–283 (66–1,526)]	< 0.001		
Operator confidence score	2 [1–2 (1–3)]	2 [2–3 (1–3)]	0.008		
FVC	n = 41	n = 33			
First-puncture success	28 (68.3%)	30 (90.9%)	0.019	1.331	1.053–1.684
First-puncture single-pass catheterization success	28 (68.3%)	30 (90.9%)	0.019	1.331	1.053–1.684
Successful final catheterization	41/41 (100%)	33/33 (100%)	-		
Puncture attempts (n)	1 [1–2 (1–4)]	1 [1–1 (1–3)]	0.029		
Puncture time	120 [52–248 (25–780)]	56 [34–82 (7.9–1,578.6)]	0.001		
Total catheterization time	340 [246–499 (130–944)]	228 [193–306 (66 –1,669)]	< 0.001		
Operator confidence score	2 [2–2 (1–3)]	3 [2–3 (1–3)]	< 0.001		

*Fisher’s Exact Test

*IJVC* Internal jugular vein catheterization; *FVC* Femoral vein catheterization; *RR* Relative risk, *CI* Confidence interval

The incidence of immediate complications was lower in the patients undergoing x-plane-guided catheterization [10/95 (10.5%) and 3/33 (9.1%) for IJVC and FVC, respectively] compared with those undergoing single-plane catheterization [25/87 (28.7%); RR, 0.366; 95% CI, 0.187–0.718; *P* = 0.002 and 14/41 (34.1%); RR, 0.266; 95% CI, 0.083–0.849; *P* = 0.011 for IJVC and FVC, respectively].

**Fig. 3:**
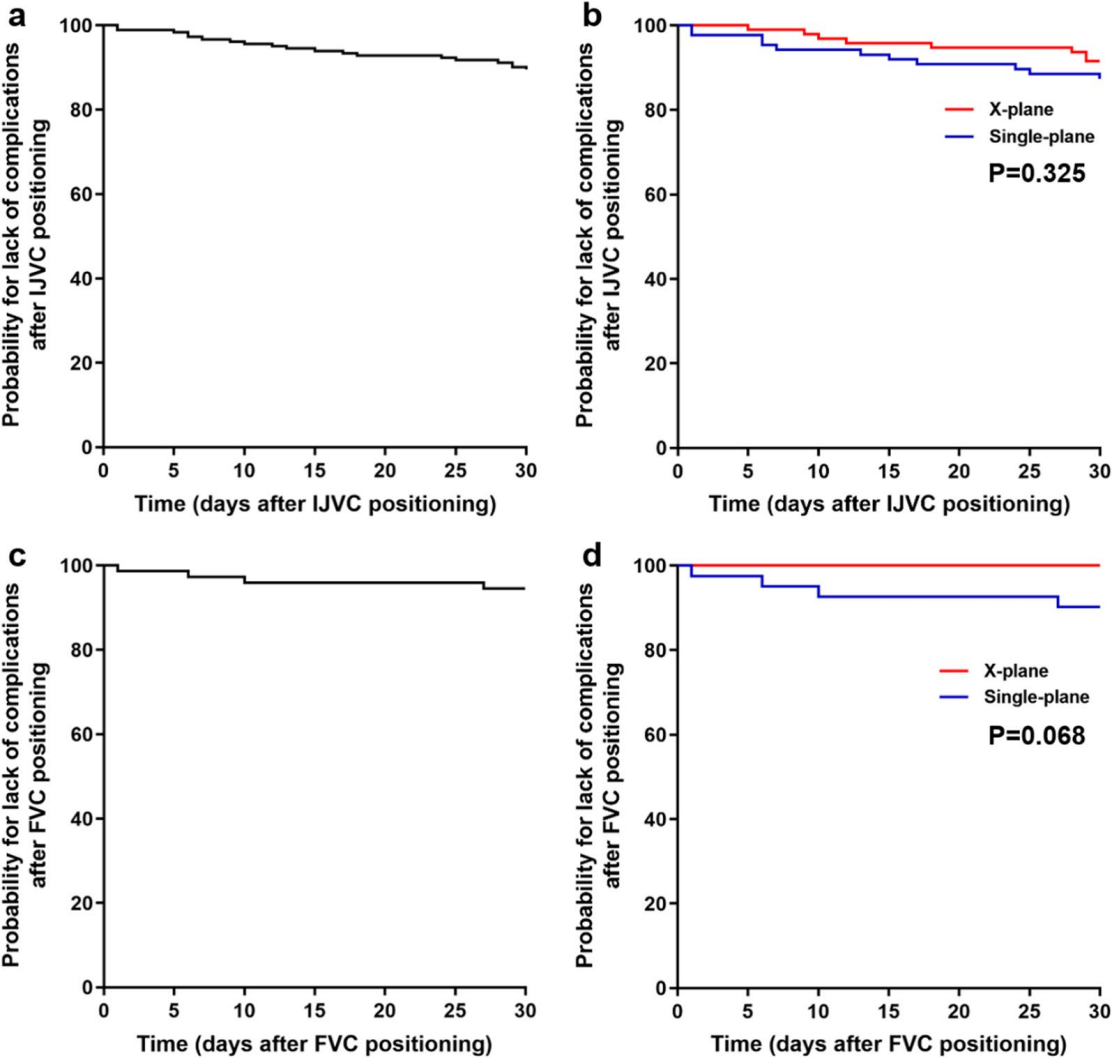
**a**, **c** Kaplan–Meier plot for the overall probability for lack of complications after internal jugular vein cannulation (IJVC) and femoral vein cannulation (FVC) in patients enrolled in the study. **b**, **d** Comparison of the probability for lack of complications after IJVC and FVC in the single-plane versus x-plane group by using the log-rank test

No statistically significant differences were observed in late complications [*P* = 0.352 and *P* = 0.124 for IJVC and FVC, respectively (Table 3)] and the Kaplan–Meier plots for the cumulative complications between the two groups [*P* = 0.325 and *P* = 0.068 for IJVC and FVC, respectively (Fig. 3)]. Specifically, 11 thromboses and four central line-associated blood stream infections (CLA-BSIs) occurred in the single-plane group compared with three thromboses in the x-plane group.

**Table 3:** Catheterization-related complications in patients in the single-plane and x-plane groups

	Single-plane (n = 128)	x-Plane (n = 128)	*P*	RR	95% CI
IJVC	n = 87	n = 95			
Immediate complications, n (%)	25 (28.7%)	10 (10.5%)	0.002	0.366	0.187–0.718
Undesired puncture	22 (25.3%)	8 (8.4%)	0.002	0.333	0.157–0.709
Hematoma	7 (8.0%)	3 (3.2%)	0.198*	0.392	0.105–1.470
Posterior IJV wall puncture	8 (9.2%)	4 (4.2%)	0.176	0.458	0.143–1.467
Arterial puncture	0	0	-		
Pneumothorax	0	0	-		
Hemothorax	0	0	-		
Late complications, n (%)	11 (12.6%)	8 (8.4%)	0.352	0.666	0.281–1.579
Venous thrombosis	9 (10.3%)	3 (3.2%)	0.051	0.305	0.085–1.091
CLA-BSI	2 (2.1%)	0	0.227*		
FVC	*n* = 41	*n* = 33			
Immediate complications, n (%)	14 (34.1%)	3 (9.1%)	0.011	0.266	0.083–0.849
Undesired puncture	11 (26.8%)	2 (6.1%)	0.020	0.226	0.054–0.949
Hematoma	3 (7.3%)	1 (3.0%)	0.624*	0.414	0.045–3.799
Posterior FV wall puncture	0	0	-		
Arterial puncture	5 (12.2%)	0	0.061*		
Late complications, n (%)	4 (9.8%)	0	0.124*		
Venous thrombosis	2 (4.9%)	0	0.499*		
CLA-BSI	2 (4.9%)	0	0.499*		

*Fisher’s Exact Test

*IJVC* Internal jugular vein catheterization; *FVC* Femoral vein catheterization; *CLA-BSI* Central line–associated blood stream infection, *RR* Relative risk, *CI* Confidence interval

## Discussion

This prospective, randomized clinical study revealed that CVCs with the x-plane approach yielded a significantly higher first-puncture success rate, operator confidence score, and shorter catheterization time, along with a lower incidence of immediate complications compared to the single-plane approach.

US-guided CVCs are recommended for routine use in ICU. However, there is no consensus on the application of in- or out-of-plane approaches, because each method has its own set of disadvantages. The out-of-plane technique requires users to follow the needle tip with the US probe, which is not intuitive for novice users and requires a significant learning curve because the needle is generally not visible until it traverses the axial imaging plane. Moreover, it requires the sliding or tilting of the transducer to follow the needle tip in real time and project the needle path, which is difficult to operate, particularly for novice operators [32]. At the same time, there is a significant risk of unsuspected needle penetration of critical structures, including the posterior venous wall and accompanying arteries [26], which can, result in complications such as hematoma formation, guidewire breakage, wire misplacement, or significant bleeding [30, 32, 33]. The in-plane method is recommended for CVC because in-plane needle insertion can maintain careful tracking of the needle tip, leading to more precise needle tip control [9, 20, 32]. The most difficult but essential task when using the in-plane method is to keep the needle in the same plane as the very narrow US beam, which requires a learning cycle. Meanwhile, generating such an image is difficult in a short neck or curved geometry of the groin, because it maintains the needle in the precise plane of the probe. Furthermore, the relationship between the target vessel and adjacent vital structures is lost, which may lead to mechanical complications.

Our study shows that the first-puncture success rate was higher for the x-plane approach than for the single-plane approach for both IJVC (91.6% vs. 74.7%) and FVC (90.9% vs. 68.3%), although the overall success rate for US-guided CVCs was 100%. The higher first-puncture success rate, fewer puncture attempts, and shorter catheterization time were mainly attributed to the advantages of the x-plane approach, which overcame the limitations of the single-plane approach and allowed users to visualize both the transverse and longitudinal views simultaneously.

The real-time biplane imaging technique offers visualization of the trajectory of the needle tip, which was confirmed by the long-axis view, and the confirmation of the needle tip inside the lumen, as well as the relationship of the target vessel to adjacent structures, which was offered by the short-axis view. This explains the higher first-puncture success rate (87.4% vs. 69.0% for IJVC and 90.9% vs. 68.3% for FVC, respectively) and shorter time (205 s vs. 311 s for IJVC and 228 s vs. 340 s for FVC, respectively) needed for CVCs in the x-plane group than that in the single-plane group. Meanwhile, the confidence score of the operator during the cannulation procedure was higher in the x-plane group (2 points for IJVC and 3 points for FVC, respectively), which could be another reason for the shorter catheterization time. Our results prove that the biplane approach optimizes the chances of successful cannulation and minimizes the risks to the surrounding structures.

The higher incidence of immediate complications in the single-plane approach (28.7% vs. 10.5% for IJVC and 34.1% vs. 9.1% for FVC, respectively) was mainly due to undesired punctures with multiple skin breach to redirect the needle. This is explained by the fact that the operator cannot line up the thin US beam with the entire length of the needle, and both have the midline axis of the vessel longitudinal plane. During CVC positioning, the hand of the operator can slide by a few millimeters, and the three axes can move out of alignment with each other, thus allowing visualization of the artery. In this context, information regarding the location of the artery and target vein is lost, and the continuous display of the needle leads to a misleading sense of safety that may ultimately lead to redirection and repuncture of the needle, as well as arterial puncture. This also caused the high rate of arterial puncture when using single-plane approach for FVC. The biplane approach allows for the visualization of the needle path in the long-axis view, as well as the needle tip, target vessel, and surrounding structures in the short-axis view, as long as the probe is positioned perpendicular to the course of the vessel. Even if the needle path cannot be clearly displayed in the long-axis view, the position of the needle tip can be visualized in the short-axis view to ensure that the needle is inserted into the vascular lumen. Using this technique, the needle tip can be observed directly, and its progression can be inferred from the movements of the surrounding tissues, even if minimal probe adjustments are required in rare cases. This could be the reason why a higher rate of first-puncture success and a shorter insertion time were achieved in the biplane approach than those in the single-plane approach in the present study.

Previous researches have reported the efficacy and safety of US-guided venous catheterization using the biplane approach [28, 34, 35]. A study using a simulated vascular model [35] revealed that biplane imaging can help avoid lateral deviation, reduce errors in identifying vessels, and prevent overshoots during CVCs. Similarly, another study [34] reported that the success rate was higher in the biplane group using a portable ultrasonic device, with a faster cannulation time and less backwall perforation in phantom model of peripheral vein. Our results are consistent with those of a previous study [28], which evaluated the out-of-plane and biplane approaches using a low-frequency transducer (1–5 MHz) to the IJVC in 100 patients by an experienced anesthesiologist and observed a higher first-pass success rate, fewer posterior wall punctures, and shorter puncture time when a biplane approach was employed. One reason why the procedure time was consistently shorter with the biplane technique than that with the single-plane technique because the latter requires a short-axis assessment of the vessels, followed by probe rotation in some cases [29]. However, these studies were either carried out on the phantom model or using a low-frequency transducer. To the best of our knowledge, this is the first study that comparing the long-axis and real-time biplane approaches using a high-frequency matrix probe for CVC in critically ill patients.

## Limitations

The study results were obtained by five less-experienced operators, which might lead to a high rate of immediate complications, and thus cannot be directly extrapolated to sonographers with more experience. Further studies are required to assess the necessity of the x-plane approach for operators with prior experience. Moreover, the out-of-plane approach was not employed in this study. This is attributed to the use of the US-guided in-plane approach by less-experienced operators because it can real-time display the needle in-plane to ensure safety. In addition, subclavian vein was not included in this study because catheterization here is associated with higher mechanical complications and high technical difficulty which is difficult for less-experienced sonographers. Further studies including experienced operators and subclavian vein catheterization are necessary to confirm our findings.

## Conclusion

In critically ill patients, the US-guided CVC x-plane approach, when performed by a less experienced operator, shows some clinical advantages, such as a higher first-puncture success rate and fewer complications, with more confidence in the procedure. Moreover, the catheter insertion time was shorter in the x-plane approach than that in the single-plane approach.

### Supplementary Information

**Additional file 1**. **Video S1.** A 78-year female underwent the internal jugular vein catheterization using the x-plane approach.

**Additional file 2: Table S1**. Ultrasonography characteristics of the study population.

## Data Availability

The datasets used and/or analyzed during the current study are available from the corresponding author on reasonable request.

## Abbreviations

CVC: Central venous catheterization
ICU: Intensive care unit
3D: Three-dimensional
US: Ultrasound
IJVC: Internal jugular catheterization
FVC: Femoral vein catheterization
RR: Relative risk
CI: Confidence interval
IQR: Interquartile range
