# Supplementary material for: Single-plane versus real-time biplane approaches for ultrasound-guided central venous catheterization in critical care patients: a randomized controlled trial
Source: Crit Care. 2023 Sep 23;27:366. doi: 10.1186/s13054-023-04635-y (PMC10517529; doi:10.1186/s13054-023-04635-y)
Supplement: Supplementary file 2 — Additional file 2: Table S1. Ultrasonography characteristics of the study population. [file 13054_2023_4635_MOESM2_ESM.docx]

Appendix Table. Ultrasonography characteristics of the study population

| Characteristics | Single-plane (n=128) | x-Plane (n=128) | *p* |
| --- | --- | --- | --- |
| IJV | n=87 | n=95 |  |
| Depth (cm) | 0.92 ± 0.28 (0.41–1.82) | 0.96 ± 0.23 (0.33–1.90) | 0.082 |
| Transverse diameter of IJV (cm) | 1.56 ± 0.47 (0.36–3.09) | 1.45 ± 0.48 (0.56–2.77) | 0.064 |
| Anteroposterior diameter of IJV (cm) | 0.95 ± 0.27 (0.20–1.64) | 0.89 ± 0.29 (0.31–1.58) | 0.093 |
| Ratio of transvers and anteroposterior diameter of IJV | 1.75 ± 0.70 (0.90–5.67) | 1.74 ± 0.66 (0.50–3.68) | 0.926 |
| Left– right diameter of CCA (cm) | 0.81 ± 0.13 (0.53–1.29) | 0.79 ± 0.11 (0.57–1.24) | 0.167 |
| Anteroposterior diameter of CCA (cm) | 0.76 ± 0.13 (0.40–1.11) | 0.73 ± 0.11 (0.52–1.03) | 0.056 |
| CCA overlap (cm) | 0.38 ± 0.27 (0–0.94) | 0.40 ± 0.27 (0–0.91) | 0.751 |
| The filling degree of IJV |  |  |  |
| Good | 66 (75.9%) | 56 (58.9%) | 0.051 |
| Fair (IJV shrinkage when slightly press the probe) | 11 (12.6%) | 19 (20.0%) |  |
| Poor (venous lumen hardly visible) | 10 (11.5%) | 20 (22.2%) |  |
| The overlap condition of IJV and CCA |  |  |  |
| Parallel | 17 (19.5%) | 10 (10.5%) | 0.188 |
| Overlap ≤50% diameter of CCA | 24 (27.6%) | 25 (26.3%) |  |
| Overlap >50% diameter of CCA | 46 (52.9%) | 60 (63.2%) |  |
| FV | n=41 | n=33 |  |
| Depth (cm) | 1.58 ± 0.56 (0.55–3.35) | 1.78 ± 0.51 (0.82–3.08) | 0.058 |
| Transverse diameter of FV (cm) | 1.10 ± 0.39 (0.57–2.69) | 1.14 ± 0.28 (0.77–1.84) | 0.322 |
| Anteroposterior diameter of FV (cm) | 0.80 ± 0.24 (0.38–1.37) | 0.88 ± 0.25 (0.52–1.44) | 0.175 |
| Ratio of transvers and anteroposterior diameter of FV | 1.43 ± 0.50 (0.69–3.09) | 1.26 ± 0.39 (0.77–3.00) | 0.097 |
| Left-right diameter of FA (cm) | 0.83 ± 0.18 (0.52–1.24) | 0.80 ± 0.19 (0.41–1.29) | 0.408 |
| Anteroposterior diameter of FA (cm) | 0.73 ± 0.18 (0.45–1.35) | 0.67 ± 0.14 (0.33–0.97) | 0.183 |
| FA overlap (cm) | 0.17 ± 0.21 (0–0.65) | 0.13 ± 0.16 (0–0.51) | 0.484 |
| The turgidity of FV |  |  |  |
| good | 20 (48.8%) | 17 (51.5%) | 0.954 |
| Fair (FV shrinkage when slightly press the probe) | 20 (48.8%) | 15 (45.5%) |  |
| Poor (venous lumen hardly visible) | 1 (2.4%) | 1 (3.2%) |  |
| The overlap condition of FV and FA |  |  |  |
| Parallel | 16 (39.0%) | 11 (33.3%) | 0.533 |
| Overlap ≤50% diameter of FA | 15 (36.6%) | 10 (30.3%) |  |
| Overlap >50% diameter of FA | 10 (24.4%) | 12 (36.4%) |  |

IJV, internal jugular vein; CCA, common carotid artery; FV, femoral vein; FA, femoral artery
